# Antiviral Drug Discovery

**DOI:** 10.3390/ijms25137413

**Published:** 2024-07-06

**Authors:** Zhenzhen Zhou, Xinyong Liu, Dongwei Kang

**Affiliations:** Department of Medicinal Chemistry, Key Laboratory of Chemical Biology, Ministry of Education, School of Pharmaceutical Sciences, Cheeloo College of Medicine, Shandong University, 44 West Culture Road, Jinan 250012, China; zhouzhensdu@163.com

A vast and painful price has been paid in the battle against viruses in global health. The COVID-19 pandemic is a stark reminder of the urgent need for antiviral agents, which provide an essential weapon for humans to overcome viral infections [1]. The substantial evidence includes that HIV-1 antivirals have successfully transformed a highly lethal disease into a manageable chronic disease [2,3,4], and influenza antivirals have been proven to shorten the duration and reduce mortality rates [5,6,7,8,9]. However, people often forget the hardships brought by a pandemic, just like forgetting the pandemic of SARS-CoV-1 in 2003 [10]. Until now, only a few viruses known to infect humans gained FDA-approved drugs for their epidemic [11]. However, viruses mutate rapidly under drug selection pressure, significantly reducing existing drugs’ therapeutic effects. Therefore, dealing with emerging viruses and combating drug resistance are significant challenges in discovering antiviral drugs.

This Special Issue “Antiviral Drug Discovery” aims to provide a fascinating landscape for the important and urgent research topics aimed at addressing the existing drug resistance and the potential emergence of new viral infections in the future. This covers some of the most important viruses, including SARS-CoV-2, human immunodeficiency virus 1 (HIV-1), influenza A virus (IAV), and avian infectious bronchitis virus (IBV). In addition, the broad-spectrum antiviral agents targeting host factors are also discussed in this issue.

In silico studies in SARS-CoV-2 antiviral drug discovery. Compared with the time-consuming and costly methods of traditional drug development, in silico computer screening can help us identify the hit compounds more effectively [12,13,14,15,16,17]. Mushebenge et al. presented a comprehensive review about the useful contributions of in silico studies in the discovery of SARS-CoV-2 receptors, highlighting some experimental validation to ensure the potency and anti-resistance profiles of the screened drug candidates [18]. Using deep learning generative models, Andrianov et al. identified seven promising SARS-CoV-2 inhibitors with novel scaffolds [19]. There is an ancient proverb in China that goes, “Teaching people to fish is better than teaching them to fish”, which emphasizes the importance of methods. The approach developed by Andrianov provides a reference for discovering novel compounds for the possible epidemic and pandemic preparedness.

HIV-1 antiviral drug discovery targeting HIV-1 RT and capsid protein. Although the highly active antiretroviral therapy (HAART) has effectively reduced the mortality of acquired immunodeficiency syndrome (AIDS) [20,21], increasing drug resistance problems force us to design a novel anti-HIV-1 drug with new targets and mechanisms. Zhou et al. presents a proof of concept for the design of a covalent inhibitor targeting HIV-1 RT to improve drug resistance profiles [22]. The inhibitor ZA-2 was demonstrated to covalently bind to Tyr318 in the HIV-1 non-nucleoside reverse transcriptase inhibitors binding pocket (NNIBP) with EC_50_ ranging from 11 to 246 nM, being far superior to that of the marketed NVP and EFV. Akther et al. conducted the structure-based design targeting HIV-1 capsid protein with the recently FDA-approved CA inhibitor GS-6207 as the lead [23]. Significantly, they work out a general synthetic route to allow the modular synthesis of novel GS-6207 subtypes, which could contribute to the further design and synthesis of novel compounds with improved potency.

IAV antiviral drug discovery targeting the Hemagglutinin 1-Mediated Virus Attachment. Since the 20th century, several outbreaks of influenza pandemics have left painful memories in human history, such as the most terrifying “Spanish Flu” in 1918–1919, killing approximate 50 million people; the “Asian Flu” in 1957–1958; the “Hong Kong Flu” in 1968–1969; the “Swine Flu” in 1976; as well as the “Russian Flu” in 1977, which also led to millions of deaths [24,25,26]. Therefore, the IAV antiviral drugs are significant as reserves for pandemic preparedness. Yang et al. identified ginsenoside G-rk1 and G-rg5 by the screening of 23 ginsenosides, which exhibited antiviral effects against 3 IAV subtypes (H1N1, H5N1, and H3N2) in vitro [27]. More importantly, they characterized the interactions between G-rk1 and HA1 in a dose-dependent manner in a surface plasmon resonance (SPR) analysis, which provided a novel method for the identification of IAV inhibitors.

The mechanism of baicalin inhibiting IBV. IBV mainly attacks birds, including broilers, which threatens the broilers’ breeding, leading to enormous cost for the global poultry industry [28,29,30]. Feng et al. demonstrated that baicalin could restore the respiratory microbiota dysbiosis and modulate amino acid metabolism to fight against IBV, which would contribute to the development of antiviral drugs against IBV [31].

Evaluation of interactions between antivirals and the immune system in antiviral drug discovery. The emergence or re-emergence of viruses with epidemic or pandemic potential, such as Ebola, SAR-CoV-1, and SAR-CoV-2, has repeatedly reminded us of the importance of developing broad-spectrum antiviral drugs [32]. The rocaglates (silvestrol, zotatifin, etc.) targeting host factors are broad-spectrum antiviral agents and efficiently inhibit the replication of Corona-, Flavi-, Picorna-, Filo- and Togaviruses by blocking viral protein synthesis [33,34,35,36,37,38,39]. Unfortunately, targeting host factors also comes with possible pleiotropic side effects [40]. Schiffmann et al. demonstrated that the inhibition of eukaryotic translation initiation factor 4A (elF4A) mRNA helicase by rocaglates could reduce the M1 MdM and T-cell and B-cell activation [41]. This interesting finding serves as a warning to the preclinical characterization of antiviral drugs that their interactions with immune system should be studied.

In summary, this Special Issue compares review and research articles on antiviral drug discovery, mainly focusing on the discovery of antivirals targeting SARS-CoV-2, HIV-1, IAV, and IBV, as well as the study of the interactions between antivirals and the immune system.

We would like to thank all the authors for their admirable work and all the reviewers for their careful evaluation of the manuscripts in this Special Issue. Finally, we sincerely appreciate the contributions of the journal editors and staff for their continuous support.
